# Concussion management knowledge among residents and students and how to improve it

**DOI:** 10.2217/cnc-2017-0001

**Published:** 2017-08-03

**Authors:** Mohammad N Haider, John J Leddy, John G Baker, John M Kiel, Michael Tiso, Karl A Ziermann, Barry S Willer

**Affiliations:** 1 Department of Psychiatry, Jacobs School of Medicine, SUNY at Buffalo, NY, 14214, USA; 2 Department of Orthopedics & Sports Medicine, Jacobs School of Medicine, SUNY at Buffalo, NY, 14214, USA; 3 Department of Nuclear Medicine, Jacobs School of Medicine, SUNY at Buffalo, NY, 14214, USA; 4 Department of Emergency Medicine, Jacobs School of Medicine, SUNY at Buffalo, NY, 14214, USA; 5 Department of Internal Medicine & Sports Medicine, Ohio State University, Columbus, OH, 43210, USA; 6 Department of Sports Medicine, Wilmington Health Primary Care, Jacksonville, NC, 28546, USA

**Keywords:** concussion, education intervention, mild traumatic brain injury, resident knowledge, teaching methods

## Abstract

**Aim:**

Recognition and management of concussion is an area of growing importance. The objective was to measure concussion knowledge among residents and medical students (MS).

**Methods:**

Baseline knowledge was assessed by a standardized questionnaire. Control group (family medicine [FM], pediatric medicine [PM] and emergency medicine) residents were given reading material, and intervention group rotated in a clinic (sports medicine residents and MS). Subjects were retested after 36.82 (16.1) days. Pre- and post-intervention test scores were compared.

**Results:**

The average baseline knowledge scores were 79.2% for emergency medicine residents, 61.4% for FM, 68.5% for PM, 71.7% for sports medicine residents and 68.0% for MS. Knowledge increase for control group was 1.16% compared with 14.41% for the clinical rotation group (p < 0.0001).

**Conclusion:**

PM and FM residents can benefit from more focused education about concussion.

Concussion, a type of mild traumatic brain injury caused by blunt trauma to the head resulting in temporary symptoms, is the most common type of traumatic brain injury [1]. It is an area of growing interest in the medical community and among researchers and increasingly is seen as a public health priority [2,3]. The incidence of concussions is increasing, which may reflect better awareness and diagnosis of the disease [4]. Sport-related concussion is a common occurrence in high school, college and professional sports [5,6]. There are an estimated 1.8–3.8 million sport-related traumatic brain injuries in the USA annually, many of which may be undiagnosed [7]. While sport-related concussion is considered to be a mild form of brain injury, it nevertheless may adversely affect quality of life [8–10]. Knowledge and recognition of concussions by physicians are critical factors in secondary prevention of postconcussive sequelae such as persistent postconcussive symptoms (PPCS) and the rare but deadly second impact syndrome [11,12].

It is important that medical providers acquire more knowledge about concussions since Meehan *et al.* [13] showed that 30.5% of patients who came to a concussion clinic had a previously undiagnosed concussion. Concussion awareness programs for coaches and athletic trainers are well established [14,15] but the lack of research on concussion knowledge transfer to residents suggests that awareness programs are limited. In 2003, 24% of US pediatric residency programs did not include formal concussion education in their curriculum [16]. A 2012 study found that a significant number of medical students (MS) and neurology and neurosurgery residents had deficient knowledge about concussion diagnosis and management [17]. In a study of Canadian medical colleges, only 29% taught their students concussion-specific knowledge [18] and the majority of American medical students reported never having a lecture dedicated to concussion during medical school or any clinical experience with concussion management [19]. In a cross-sectional study by Stoller *et al.* [20], 27–52% of pediatricians, emergency room doctors and family physicians had no knowledge of international consensus guidelines on concussion management.

Since studies have identified important gaps in physician knowledge about concussion, the primary aim of this study was to rigorously identify baseline knowledge among different residency specialties (family medicine [FM], pediatric medicine [PM] and emergency medicine [EM]) residents and to assess the efficacy of a focused education compared with passive teaching though written material as a method of knowledge transfer. The rational for including these specific residency programs was that they likely have more exposure to concussed patients; hence, the need for baseline knowledge is important. The secondary aim was to examine the sources of concussion knowledge for physicians and MS.

## Methods

To assess medical knowledge among residents, we approached them with a survey during their respective grand rounds along with a brief explanation of the study. For MS, only those who were going to rotate in sports medicine (SM) were asked to participate at the beginning of their rotation. Those who agreed to participate were given the preintervention test on the same day. The study was approved by the university institutional review board.

### Participants

We separated the participants into a total of five subgroups: EM residents; PM residents; FM residents not rotating through the SM department; internal medicine residents rotating through SM department; and MS. Residents in EM, PM and FM constituted the control group and SM residents and MS constituted the clinical rotation group. SM and MS were asked to participate in their first grand rounds conference, 2 days after the start of their rotation, to minimize the effect of departmental rotation. First-, second- and third-year residents were eligible to participate. The participants were given a $10 gift card if they completed both the pre- and post-intervention tests.

### Survey instrument

The survey was composed of two parts. The first part consisted of 23 multiple choice questions that tested general knowledge about concussions. This questionnaire was developed by senior clinicians who specialize in concussion management in the Concussion Clinic for the purpose of this study. The content of the questions were from the list of reading material provided to the control group. The questions were divided into three categories: 12 questions about diagnosis, seven questions about treatment and four questions about current guidelines.

The second part of the test included six questions that inquired about how the students/residents learned about concussions. The questions inquired about the source of their learning during medical school and residency and if they had clinically observed or managed a patient with acute concussion or PPCS. Although the surveys given to residents and MS were identical, the MS were asked to ignore questions in the second part relating to experience during residency.

### Intervention

There were two types of intervention. The control group received usual residency training and was given a short list of reading materials providing overviews of concussion and current concussion management guidelines. The literature included the following: 2012 Zurich Guidelines [21], Management of Concussion and Post-Concussion Syndrome [22], Importance of Return to Learn in Pediatric and Adolescent Concussion [23] and a handout on the physical examination for concussion. The purpose of giving the control group literature was to control for the effect of being part of an intervention and the expectation that the intervention will have an effect. The clinical rotation group was given the same reading material and participated in 2 half-day concussion clinic sessions per week for 4 weeks. Each day, the clinic sees between 12 and 15 acute concussions or individuals with PPCS. The clinical rotation group was tested within the first 2 days of the start of their rotation to minimize the effects of clinical experience during the Concussion Clinic rotation. All participants were retested using the same test approximately 1 month later.

### Statistical analysis

We analyzed data using SPSS Version 20 (IBM Corporation, NY, USA). The total score from the first and second tests was converted to a percentage. Descriptive statistics were used to analyze knowledge scores of the four groups of physicians and MS. The scores were also categorized into diagnosis, treatment and guidelines, and were presented separately for each group. The change in knowledge scores was calculated using repeated measures analysis of variance (ANOVA) to examine differences between interventions. Two-tailed p < 0.05 was considered to be statistically significant. A multivariate ANOVA was used to assess any difference between groups. A *post hoc* analysis with Bonferroni Correction was used to compare subgroups. Past experience of clinically managing or observing a concussion and sources of knowledge are presented in descriptive frequencies and correlation is measured using a *t*-test.

## Results

Out of 149 prospective participants, a total of 126 agreed to be part of the study but 54 (42.9%) of them were lost to follow-up (16 EM, ten FM, 22 PM and six MS). The participants who did not complete the test at the second time point were not included for the final analysis; thus, we had 72 (57.1%) participants who completed both surveys. None of the remaining surveys had missing information. The recruitment rate from total prospective participants for each subgroup was 0.33 for FM, 0.44 for PM, 0.29 for EM, 1 for SM and 0.6 for MS.

The postintervention test was completed an average of 36.8 (16.1) days later. Most of the delays were due to PM and EM residents delaying their postintervention test. There was minimal variability in the SM and MS groups in time duration of intervention. The final sample included 15 FM residents, 20 PM residents, 14 EM residents, 14 SM residents and nine MS. Table 1 shows the pre- and pos-intervention test results and the average change in scores.

**Table 1.** **Pre- and post-mean percentage survey scores for residents and medical students.**

**Department**	**Mean preintervention knowledge (%; SD)**	**Mean postintervention knowledge (%; SD)**	**Mean change in pre- and post-intervention knowledge (%; SD)**
Family Medicine^†^ (n = 15)	61.45 (16.68)	66.38 (16.47)	4.93 (18.17)

Pediatric Medicine^†^ (n = 20)	68.48 (10.82)	69.80 (10.61)	1.32 (8.25)

Emergency Medicine^†^ (n = 14)	79.21 (6.41)	76.10 (7.20)	-3.11 (10.57)

Medical Students^‡^ (n = 9)	67.98 (11.82)	78.70 (6.22)	10.72 (14.41)

Sports Medicine^‡^ (n = 14)	71.74 (10.89)	88.53 (9.89)	16.79 (12.29)

Total (n = 72)	69.67 (12.85)	75.07 (13.29)	5.39 (14.25)

^†^Indicates literature only group.

^‡^Indicates literature and clinical rotation group.

SD: Standard deviation.

As can be seen in Table 1, there was an average increase of 5.39% from pre- to post-examinations. The mean preintervention test scores of the included participants (score of 69.7%) was not statistically different from the preintervention test scores of all the participants who completed the first test (score of 72.3%; p = 0.28). A repeated measures, ANOVA was significant for the interaction between intervention group and change in test score from pre- to post-clinical experience (F = 16.5; p < 0.0001). Before intervention, both groups were similar in concussion management test scores (control group score: 69.3% vs clinical rotation group score: 70.3%; p = 0.326). The clinical rotation group scored significantly higher on the postintervention score (84.7%) when compared with the control group (70.6%; p < 0.001). Mean increase in knowledge score for the control group was 1.16% (12.78) compared with 14.41% (13.19) for the clinical rotation group. *Post hoc* analysis with Bonferroni correction revealed significant differences between some subgroups. In the preintervention test, there was a significant (p < 0.0001) difference between FM and EM residents only, with EM residents demonstrating greater knowledge. The postintervention tests, however, revealed significant differences between SM and FM (p < 0.0001), SM and PM (p < 0.0001), and SM and ED (p = 0.046). Table 2 shows the scores of the residents and students on the preintervention survey for the different categories of questions, (i.e., diagnosis, treatment and guidelines). One-way ANOVAs revealed significant differences between groups in treatment (p < 0.0001) and diagnosis (p = 0.04) but not for guidelines (p = 0.07).

**Table 2.** **Mean test-survey raw scores according to knowledge content areas.**

**Department**	**Preintervention**			**Postintervention**		

	**Diagnosis (SD; max = 12)**	**Treatment (SD; max = 7)**	**Guidelines (SD; max = 4)**	**Diagnosis (SD; max = 12)**	**Treatment (SD; max = 7)**	**Guidelines (SD; max = 4)**
Family Medicine	6.47 (2.17)	4.73 (1.22)	2.93 (1.10)	6.98 (2.33)	5.01 (1.32	3.48 (0.92)

Pediatrics	7.50 (1.19)	4.80 (1.20)	3.45 (1.00)	7.62 (1.17)	5.05 (1.42)	3.43 (1.16)

Emergency Medicine	8.29 (0.99)	6.14 (0.86)	3.79 (0.58)	7.95 (1.86)	5.93 (0.78)	3.62 (0.62)

Medical Students	7.78 (1.48)	3.89 (1.90)	3.78 (0.44)	8.64 (1.33)	5.60 (1.24)	3.86 (0.32)

Sports Medicine	7.21 (1.18)	5.93 (1.14)	3.36 (0.75)	10.06 (1.17)	6.39 (0.56)	3.91 (0.29)

Total	7.37 (1.66)	5.33 (1.27)	3.38 (0.92)	8.15 (1.72)	5.63 (1.10)	3.49 (0.48)

SD: Standard deviation.

Table 3 shows the percentage of residents who had clinically managed a patient with acute concussion or postconcussion syndrome. Comparing the effect of having clinically managed a patient with concussion on the knowledge percentage scores in the preintervention test (yes = 73.7 [10.5] vs no = 66.0 [13.8]), we see that there is a significant difference, t = -2.6; p < 0.01.

**Table 3.** **Percent and number of residents and students with patient management experience.**

**Department**	**Preintervention-managed patient (%)**	**Postintervention-managed patient (%)**
Family Medicine	13.3	26.7

Pediatrics	70.0	80.0

Emergency Medicine	92.8	92.8

Medical Students	22.2	66.7

Sports Medicine	21.4	85.8

Total	47.2	70.8

Finally, residents said they learned about concussions during residency from clinical experience (41.2%), self-study (39.9%) and lectures (27.0%). Only 19.1% said they had never learned about concussions during their residency. The residents and MS combined said that during medical school they learned about concussions from lectures (60%), emergency department clerkships (21.4%), pediatric clerkships (18.6%) and FM clerkships (17.1%). The remaining 10% of participants said that they had never learned about concussions during medical school.

## Discussion

Our results show that EM residents had the highest baseline scores for knowledge of concussion evaluation and management prior to any intervention. More than 90% of the EM residents who completed the pretest had clinically managed a patient with concussion. Residents and students said that they learned about concussions first from lectures and second during their emergency department clerkship indicating the emergency department may have more clinical exposure to concussions than the other departments. As for the paradoxical decrease in the postintervention knowledge scores of EM residents (a 3% reduction that was not statistically significant), this may have been due in part to the effect of regression toward the mean [24].

The PM and FM residents were quite similar in that they started off with average scores that did not change much after the intervention. The PM residents had slightly more knowledge than the FM residents (not significant), which may reflect the fact that 70% of PM residents versus 13.3% of the FM residents had clinically managed a concussion patient prior to the pretest. MS had slightly more concussion knowledge than the FM residents. This may be because awareness about concussions is growing each year and more emphasis is being put on teaching this topic in medical school. Unfortunately, the sample size of the MS group was too small to accurately reflect the medical school’s student population.

The results of this study support the effectiveness of focused concussion education and clinical experience at a concussion clinic compared with regular residency training in increasing knowledge of concussion management. The residents and MS who completed a clinical rotation in sports medicine (SM and MS), including training in concussion management, showed significant improvement in survey scores compared with residents who did not. This may be due to the perceived need to know about concussions during a SM rotation, which may have encouraged greater learning about concussion management. There is support in the literature for the effectiveness of active compared with passive learning. Hwang *et al.* [25] showed that active learning improved student’s performance when compared with passive learning. Freeman *et al.* [26] demonstrated that active learning was superior to passive learning in mathematics, science and engineering. This study suggests that this concept is applicable to clinical knowledge as well.

An important limitation to this study is that there was no randomization to intervention group, although a SM rotation was mandatory for all students and internal medicine residents, and therefore this group should not have been different from their peers at the onset of the concussion clinic rotation. Some subjects may have been more interested in concussions or pursuing a career in SM and so may have been more eager to learn about them. Other limitations of this study include that there are other ways, residents and MS could have learned about concussion. We could not account for any extra sources of knowledge during the intervention period for both groups nor could we confirm that the literature-only and clinical rotation groups actually read the literature we gave them. First-, second- and third-year residents were allowed to participate in the study but the difference in years of experience was not accounted for and could have been a confounder when it came to clinical experience with an acute concussion or cumulative knowledge about concussion management. Generalizability is a potential limitation. We assume that university-based programs should be able to replicate our findings because SM physicians should have adequate knowledge about concussion to be able to teach students and residents. Finally, there is potentially nonresponse bias due to the high rate of participants lost to follow-up. There was, however, no statistically significant difference in PreIntervention scores between those who completed and those who did not complete the follow-up test.

## Conclusion

Our data suggest that different clinical exposure and experience during some residency specialties (EM) impart more knowledge on concussion management when compared with others (PM and FM). The data also support the superior efficacy of an active mentored approach to transmitting knowledge about concussion management to students and residents when compared with passive methods of learning like written information about concussions.

## Future perspective

PM and FM residents would likely benefit from more clinical exposure to concussed patients.

**Summary points**

- Concussion management knowledge is lower among pediatric and family medicine residents when compared with emergency medicine residents.
- Emergency medicine residents have greater exposure to concussions than other residency specialties.
- Knowledge transfer about concussion management through a focused, mentored teaching method appears to be superior to a passive program of reading literature.
